# Supplementary material for: Plasma Levels of snoRNAs are Associated with Platelet Activation in Patients with Peripheral Artery Disease
Source: Int J Mol Sci. 2019 Nov 27;20(23):5975. doi: 10.3390/ijms20235975 (PMC6929168; doi:10.3390/ijms20235975)
Supplement: Supplementary file 1 [file ijms-20-05975-s001.pdf]

Supplemental Data

Plasma levels of snoRNAs are associated with platelet activation in patients with peripheral artery disease

Anne Yaël Nossent, Neda Ektefaie, Johann Wojta, Beate Eichelberger, Christoph Kopp, Simon Panzer, Thomas Gremmel

Curve estimations for SNORD113.2 and SNORD114.1 expression in plasma of 104 consecutive PAD patients with parameters of platelet function.

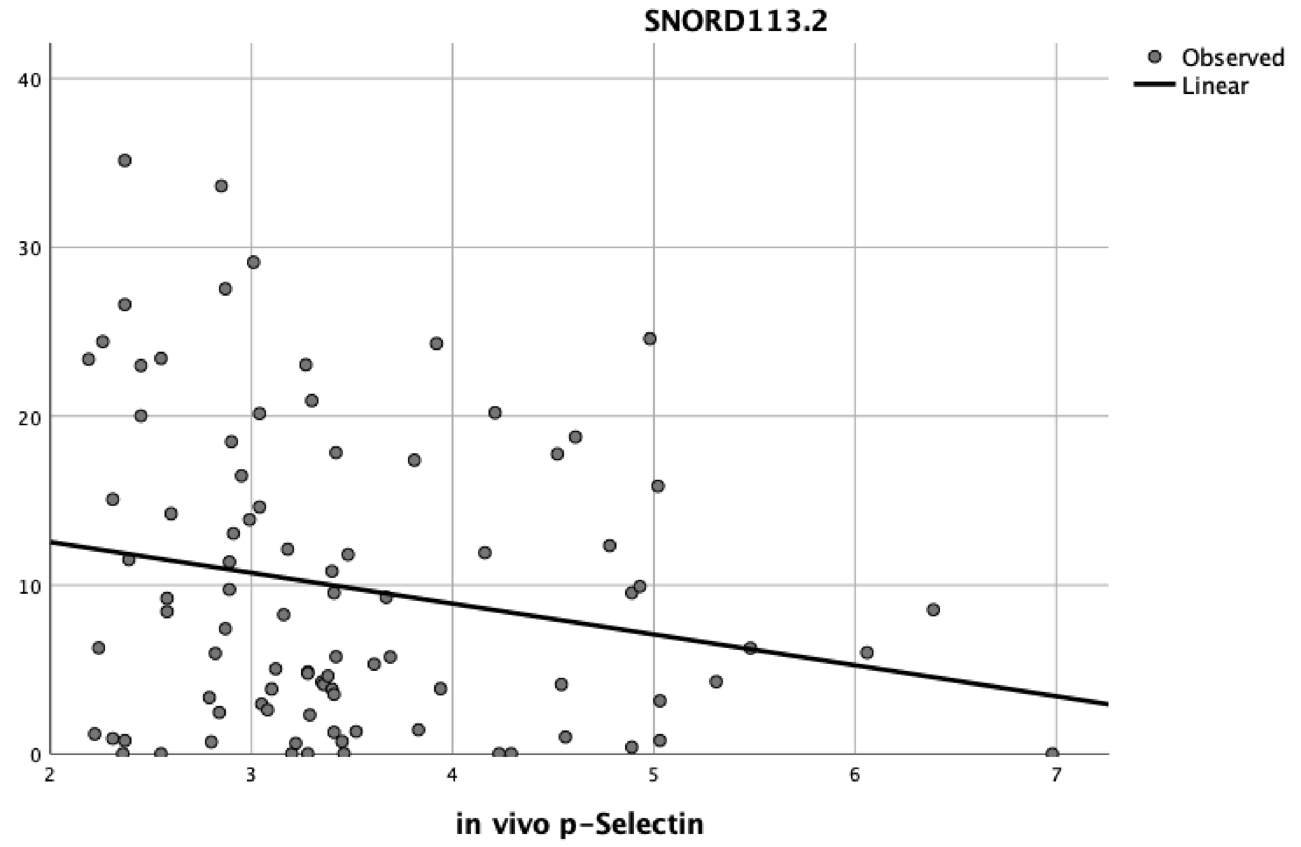
SNORD113.2
Observed
Linear
40
30
20
10
0
2
3
4
5
6
7
in vivo p-Selectin

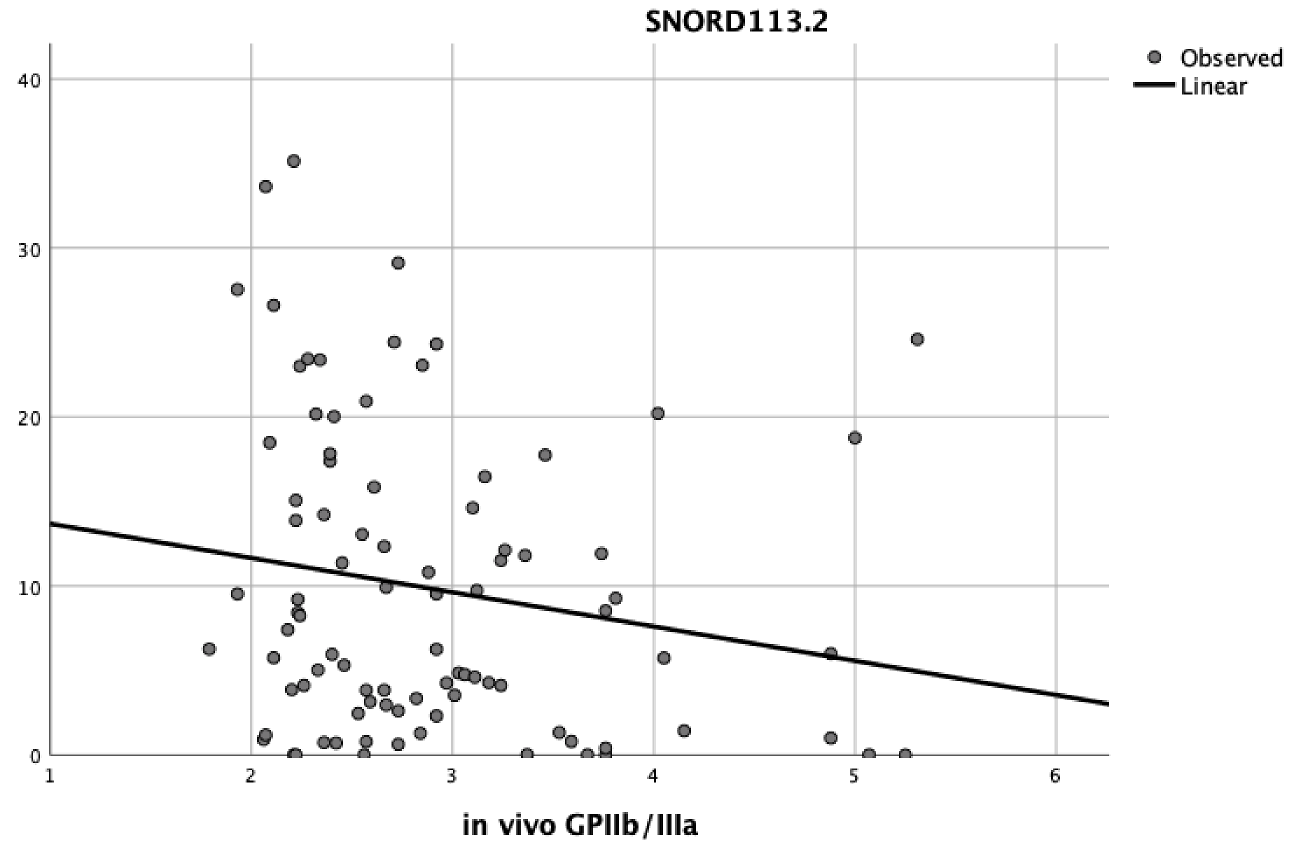
SNORD113.2
Observed
Linear
40
30
20
10
0
1
2
3
4
5
6
in vivo GPIIb/IIIa

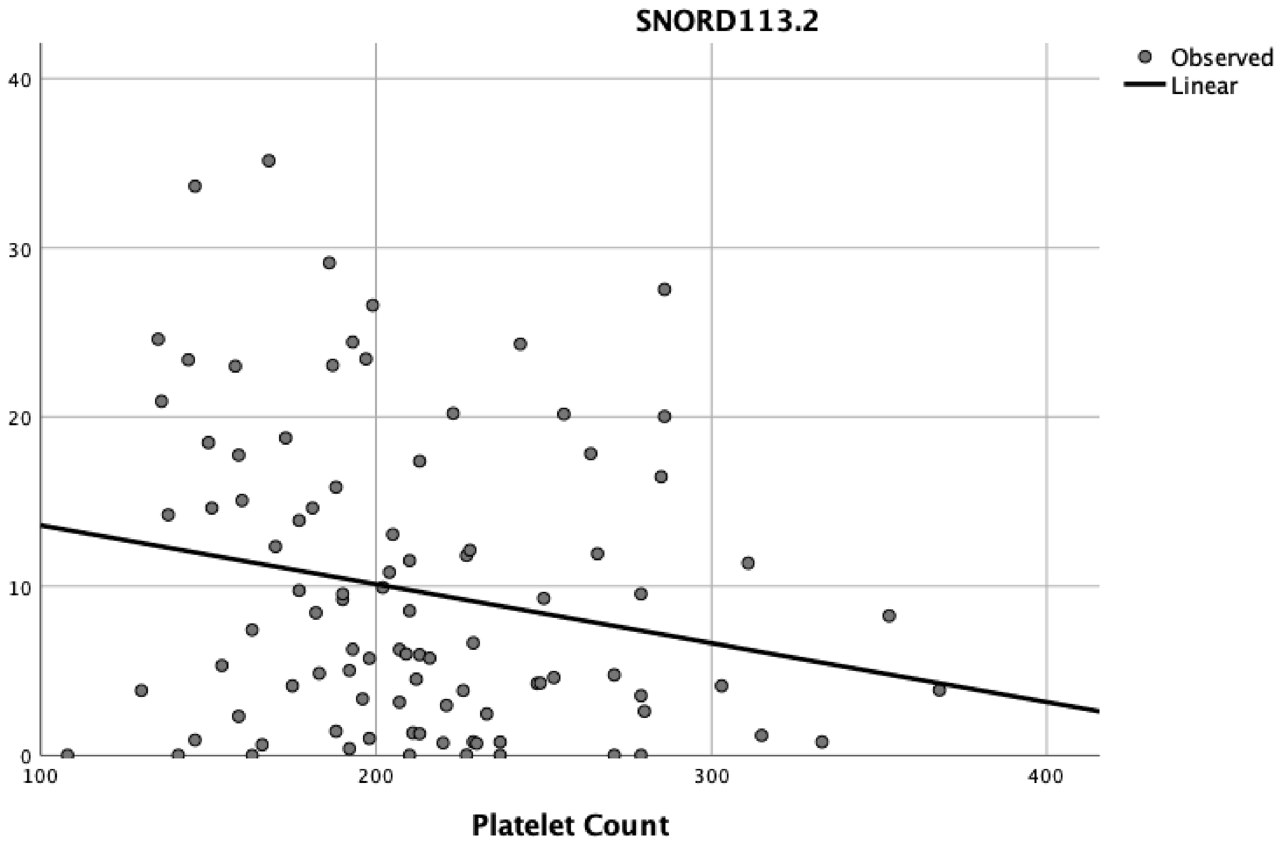
SNORD113.2
Observed
Linear
0
10
20
30
40
100
200
300
400
Platelet Count

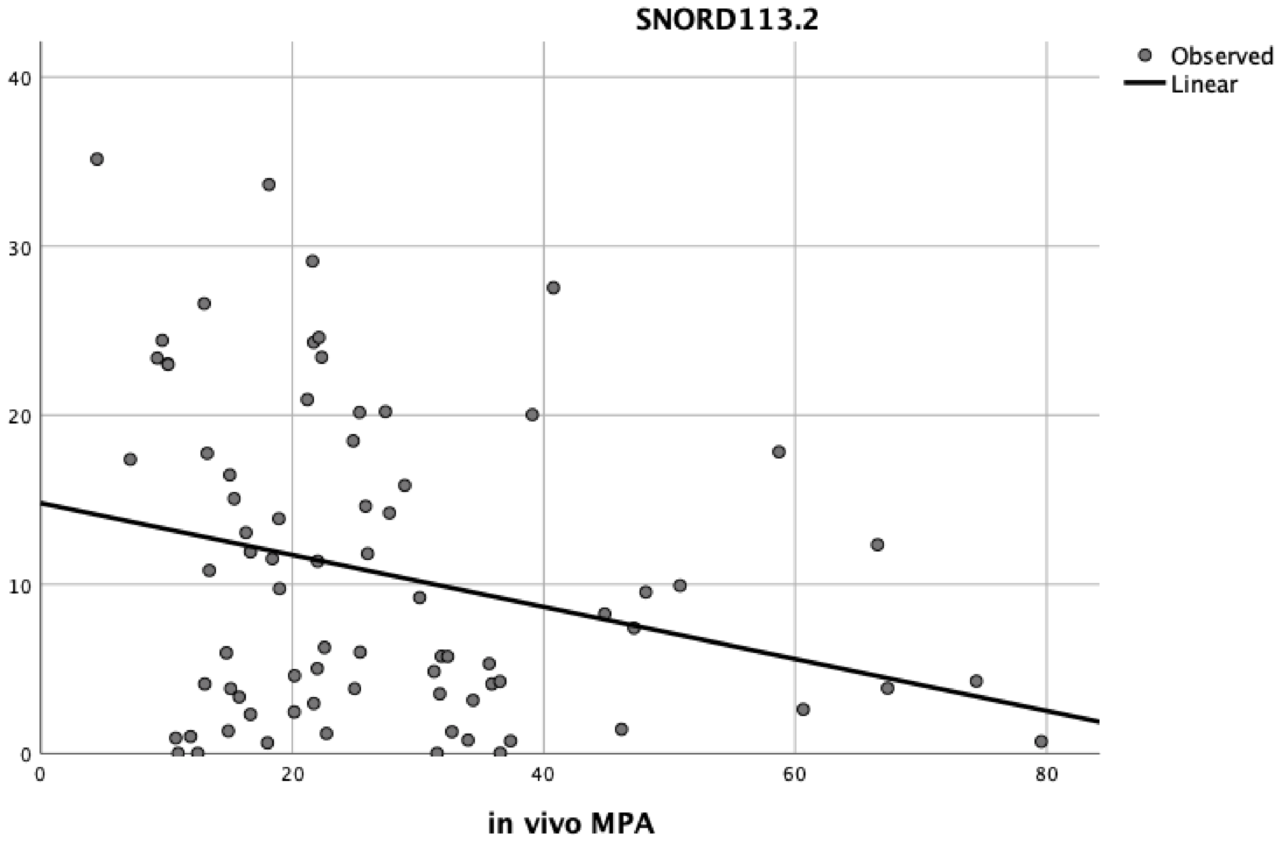
SNORD113.2
Observed
Linear
0
10
20
30
40
0
20
40
60
80
in vivo MPA

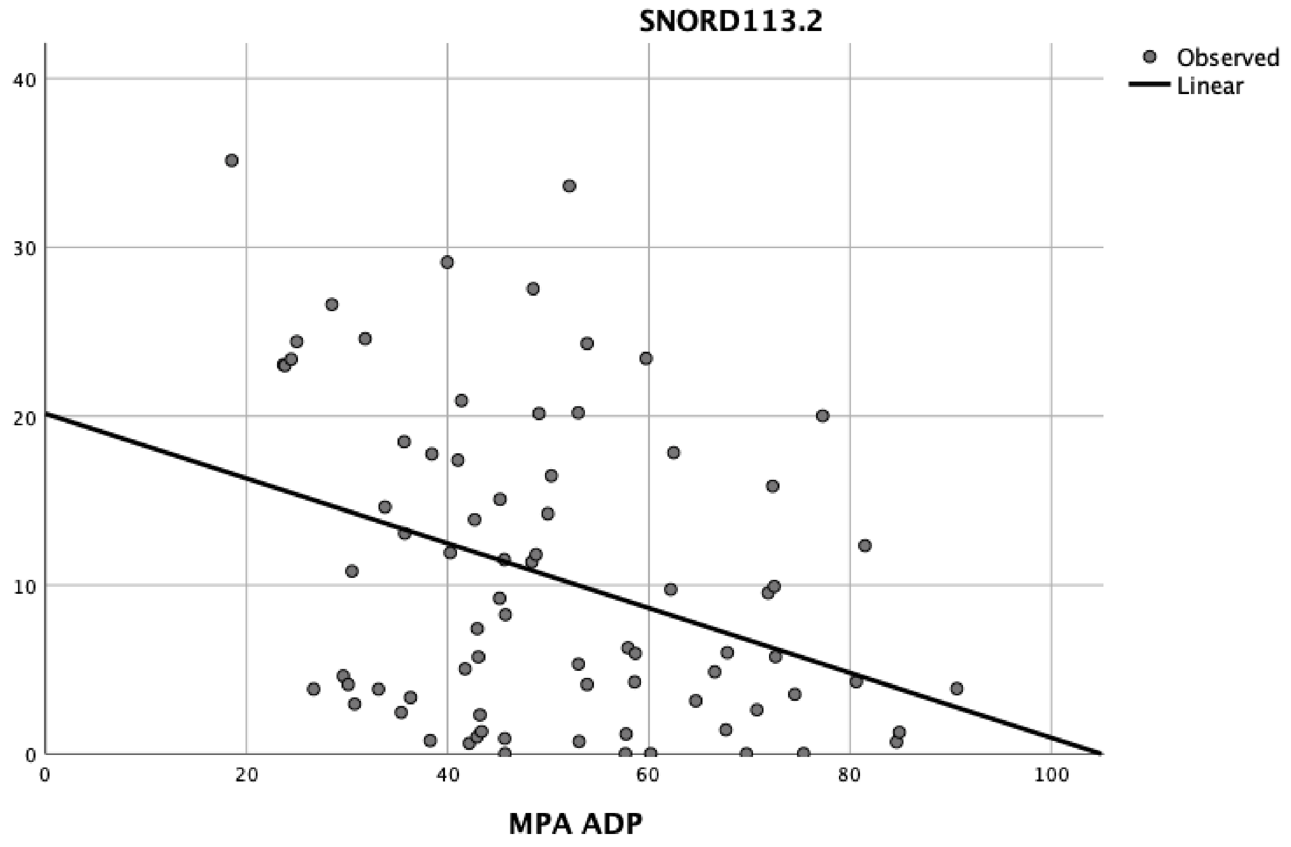
SNORD113.2
Observed
Linear
40
30
20
10
0
0
20
40
60
80
100
MPA ADP

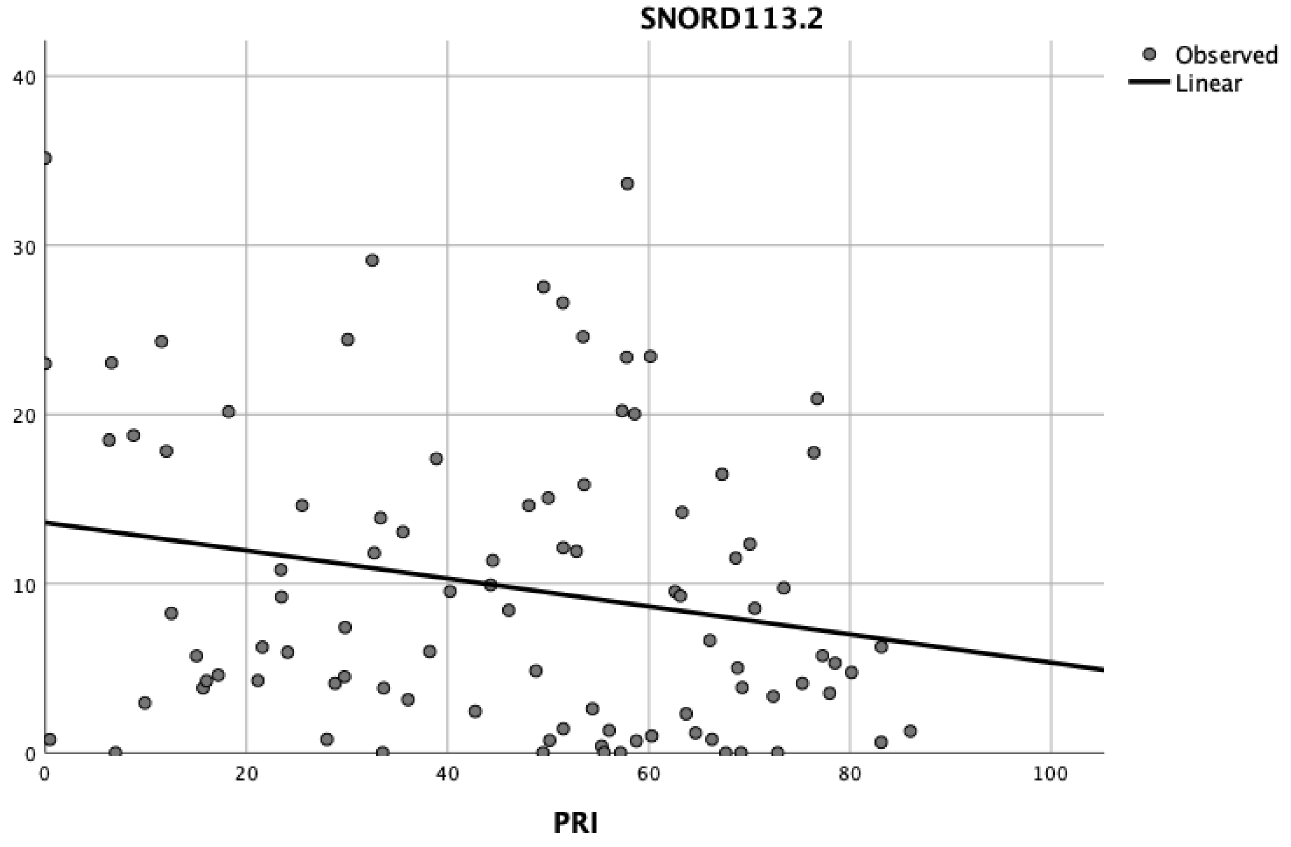
SNORD113.2
Observed
Linear
40
30
20
10
0
0
20
40
60
80
100
PRI

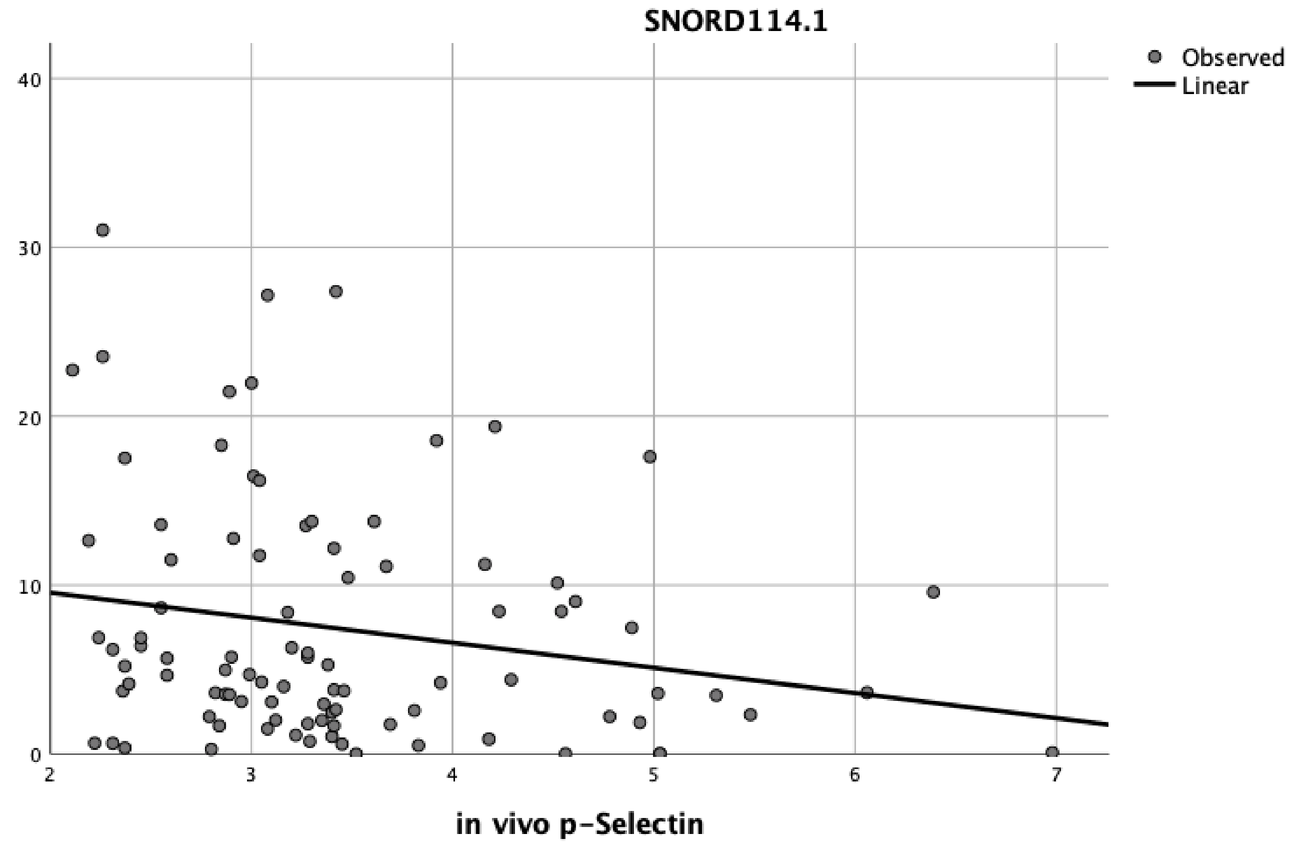
SNORD114.1
Observed
Linear
40
30
20
10
0
2
3
4
5
6
7
in vivo p-Selectin

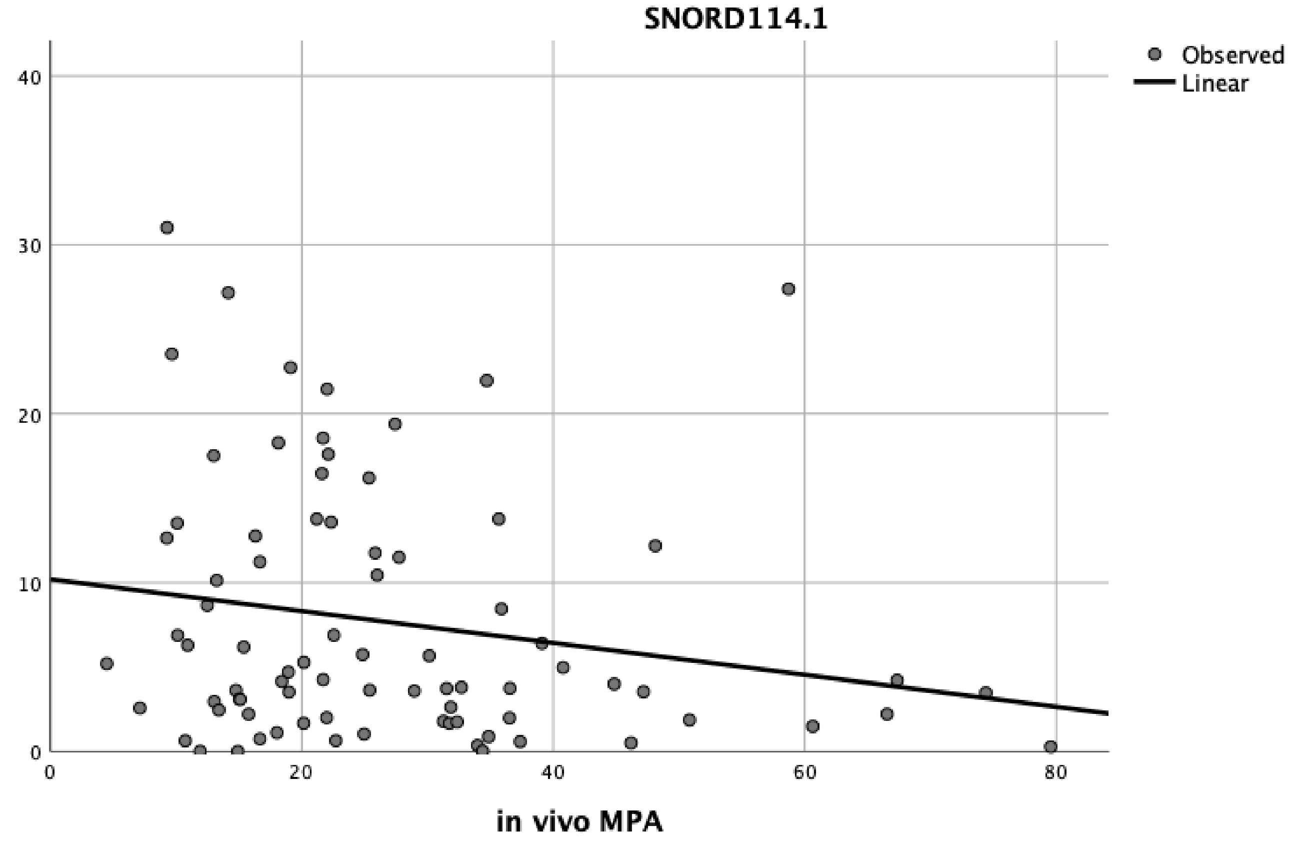
SNORD114.1
Observed
Linear
40
30
20
10
0
0
20
40
60
80
in vivo MPA

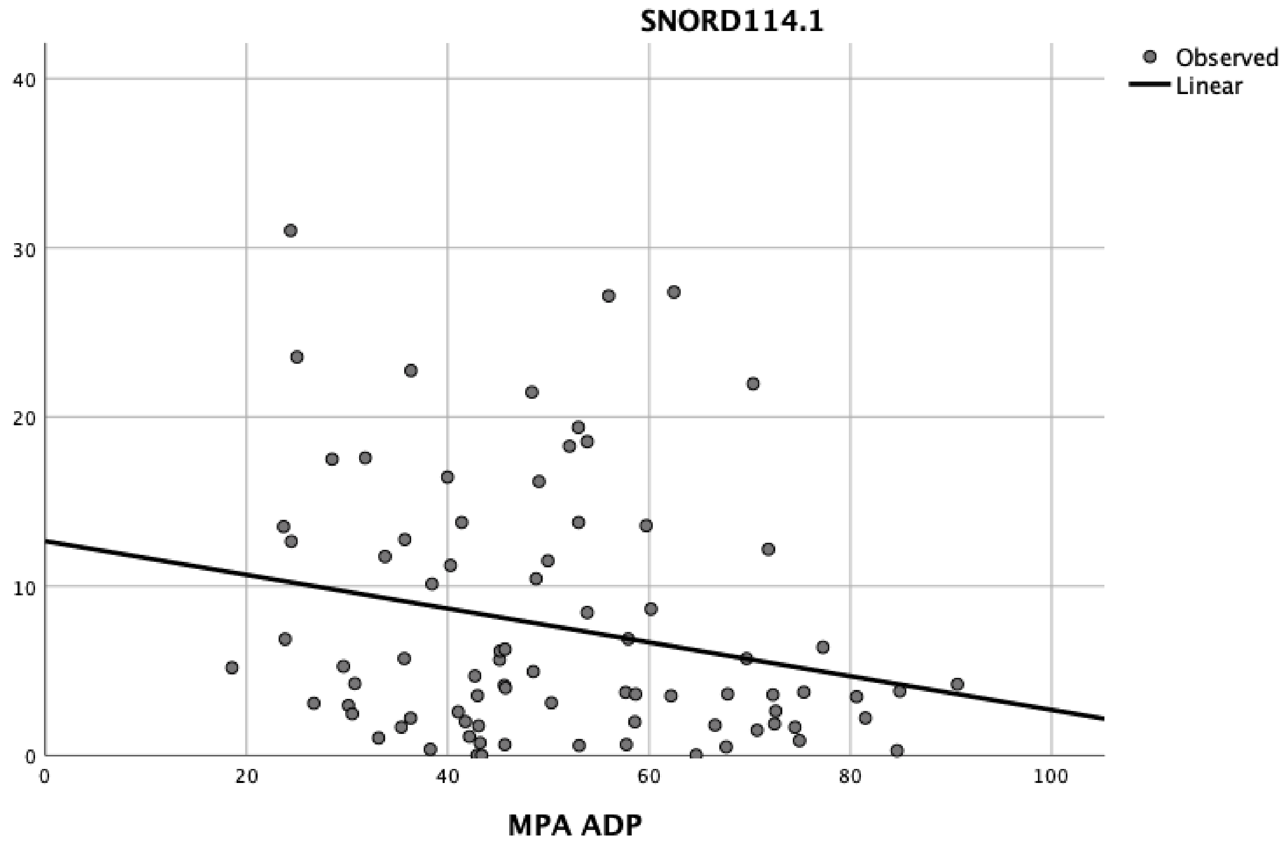
SNORD114.1
Observed
Linear
40
30
20
10
0
0
20
40
60
80
100
MPA ADP
